# Drainless robot-assisted minimally invasive oesophagectomy—randomized controlled trial (RESPECT)

**DOI:** 10.1186/s13063-023-07233-z

**Published:** 2023-05-02

**Authors:** B. Müssle, J. Kirchberg, N. Buck, O. Radulova-Mauersberger, D. Stange, T. Richter, B. Müller-Stich, R. Klotz, J. Larmann, S. Korn, A. Klimova, X. Grählert, E. Trips, J. Weitz, T. Welsch

**Affiliations:** 1 Department of Visceral, Thoracic and Vascular Surgery, University Hospital and Faculty of Medicine Carl Gustav CarusTechnische Universität Dresden, Dresden, Germany; 2 Current Address: Department of General, Visceral and Thoracic Surgery, St. Elisabethen-Klinikum Ravensburg, Academic Teaching Hospital of the University of Ulm, Ulm, Germany; 3 National Center for Tumour Diseases (NCT/UCC), Dresden, Germany; 4 German Cancer Research Center (DKFZ), Heidelberg, Germany; 5 Faculty of Medicine and University Hospital Carl Gustav Carus, Technische Universität Dresden, Dresden, Germany; 6 Helmholtz-Zentrum Dresden - Rossendorf (HZDR), 01307 Dresden, Germany; 7 Core Unit for Data Management and Analytics, National Center for Tumour Diseases (NCT), Dresden, Germany; 8 Department of Anaesthesiology and Critical Care Medicine, University Hospital Carl Gustav Carus Dresden, Technische Universität Dresden, Dresden, Germany; 9 Department of General, Visceral and Transplantation Surgery, Heidelberg University Hospital, Heidelberg, Germany; 10 Department of Anaesthesiology, Heidelberg University Hospital, Heidelberg, Germany; 11 Coordination Centre for Clinical Trials, Faculty of Medicine Carl Gustav Carus, Technische Universität Dresden, Dresden, Germany; 12 Department of General, Visceral and Thoracic Surgery, University Hospital Hamburg-Eppendorf, Hamburg, Germany

**Keywords:** RAMIE, Oesophageal cancer, Oesophagectomy, Postoperative pain, ERAS (enhanced recovery after surgery)

## Abstract

**Background:**

The purpose of this randomized trial is to evaluate the early removal of postoperative drains after robot-assisted minimally invasive oesophagectomy (RAMIE). Evidence is lacking about feasibility, associated pain, recovery, and morbidity.

**Methods/design:**

This is a randomized controlled multicentric trial involving 72 patients undergoing RAMIE. Patients will be allocated into two groups. The “intervention” group consists of 36 patients. In this group, abdominal and chest drains are removed 3 h after the end of surgery in the absence of contraindications. The control group consists of 36 patients with conventional chest drain management. These drains are removed during the further postoperative course according to a standard algorithm. The primary objective is to investigate whether postoperative pain measured by NRS on the second postoperative day can be significantly reduced in the intervention group. Secondary endpoints are the intensity of pain during the first week, analgesic use, number of postoperative chest X-ray and CT scans, interventions, postoperative mobilization (steps per day as measured with an activity tracker), postoperative morbidity and mortality.

**Discussion:**

Until now, there have been no trials investigating different intraoperative chest drain strategies in patients undergoing RAMIE for oesophageal cancer with regard to perioperative complications until discharge. Minimally invasive approaches combined with enhanced recovery after surgery (ERAS) protocols lower morbidity but still include the insertion of chest drains. Reduction and early removal have been proposed after pulmonary surgery but not after RAMIE. The study concept is based on our own experience and the promising current results of the RAMIE procedure. Therefore, the presented randomized controlled trial will provide statistical evidence of the effectiveness and feasibility of the “drainless” RAMIE.

**Trial registration:**

ClinicalTrials.gov NCT05553795. Registered on 23 September 2022.

## Background

In recent years, the incidence of oesophageal cancer has increased rapidly in the Western world [1, 2]. The 5-year overall survival among patients with oesophageal cancer is between 10 and 15%, rising to 40% after curative resection [3, 4]. Improvements in overall survival after curative oesophagectomy have been observed in recent years because of centralization to high-volume centres and the growing introduction of multimodal treatment strategies [5, 6]. Therefore, the up-to-date clinical standard for resectable oesophageal cancer is curative surgical resection, often preceded by neoadjuvant treatment protocols. The latest results of minimally invasive oesophagectomy have shown a major reduction in postoperative complications and pain compared with standard open resection [7–9]. Patients with fewer complications further benefit from an improved overall survival [10].

Robot-assisted minimally invasive oesophagectomy (RAMIE) represents a further technical improvement compared to the laparoscopic procedure. It offers improved three-dimensional visualization, magnification, and mechanized control that transforms the surgeon’s larger movements into precise robotic movements, facilitating the surgeon’s ability to perform complex surgical procedures [8]. RAMIE, with its reduced invasiveness, reduces postoperative pain and leads to a rapid and enhanced mobilization of patients. In addition, it results in fewer surgical site infections, less pulmonary impairment, and fewer complications. This leads to a faster recovery, shortens the length of hospital stay, and saves health care costs. There is evidence that disease-free and overall survival are not influenced by RAMIE. Regarding this background, currently, the minimally invasive approach should be favoured because of the lower postoperative morbidity [10].

A further advance in surgical care was the introduction and implementation of enhanced recovery after surgery (ERAS) programmes designed to reduce the physical and psychological stress of patients undergoing surgery, thereby accelerating postoperative recovery and reducing perioperative morbidity, hospital stay, and healthcare costs [11, 12]. Studies have shown that ERAS concepts can also be performed safely during oesophageal surgery. The data reflect that early postoperative mobilization of the patient, preferably on the day of surgery, dispensing with nasogastric tubes and drains contributes to a sustained impact on postoperative morbidity and hospital stay [13].

In recent years, chest drain management strategies have therefore undergone sustainable changes.

Chest drain management is also a crucial factor determining the postoperative course in patients undergoing RAMIE, influencing postoperative pain and the speed of functional recovery, and thereby the length of hospital stay. Two chest drains have traditionally been used to drain the right pleural cavity after oesophageal resections. One was placed at the apex to drain the air, while the second was placed at the recessus to drain the pleural effusion [14]. Interestingly, the incidences of postoperative pulmonary complications and thoracocentesis were not significantly different between early and late chest drain removal in patients after oesophagectomy with three-field lymph node dissection. Furthermore, early removal of chest drains supports early initial mobilization after surgery [15]

Given the above, we aim to investigate the potential advantages of RAMIE in oesophageal cancer without postoperative chest drains compared with the postoperative use of chest drains, which is considered the standard worldwide. The primary hypothesis is that avoidance of chest drains during the postoperative course after RAMIE can further reduce postoperative pain, improve functional recovery, and shorten hospital stay compared with the use of chest drains.

## Methods

### Aim of the study

This study aims to evaluate two different chest drain management strategies in patients undergoing RAMIE for oesophageal cancer with regard to perioperative complications until discharge. The study’s primary objective is to investigate whether the intensity of postoperative pain can be significantly reduced by avoiding chest and abdominal drains within 3 h after RAMIE, resulting in a drainless postoperative course. We assume that this will influence secondary endpoints such as early recovery and length of hospital stay.

### Trial design, study registration, and ethics

The title of this clinical trial is “Drainless Robot-assisted Minimally Invasive Oesophagectomy” (RESPECT Trial). The study is designed as a randomized controlled multicentric, two-arm parallel-group superiority surgical trial with an interventional group (A: robot-assisted minimally invasive oesophagectomy with early removal of the chest drain) and a control group (B: robot-assisted minimally invasive oesophagectomy with chest drains). The study is coordinated by the leading Study Center of the Department of Visceral, Thoracic and Vascular Surgery, Universitätsklinikum Carl Gustav Carus, Technische Universität Dresden, Germany.

Participating centres at the trial commencement are the two certified university centres: the Department of Visceral, Thoracic and Vascular Surgery, Universitätsklinikum Carl Gustav Carus, Technische Universität Dresden, Germany, and the Department of General, Visceral and Transplantation Surgery, University Hospital Heidelberg, Heidelberg, Germany. Additional centres can be evaluated for participation if equivalent standards for the operation (RAMIE procedure with equal technique) and trial management can be guaranteed at these sites. The trial was registered in advance at ClinicalTrials.gov (https://clinicaltrials.gov/ct2/show/NCT05553795). The study protocol was approved by the Ethical Committee at the Technische Universität Dresden (BO-EK-77022021). Any modifications to the protocol will require a formal amendment of the protocol. Amendments will be re-evaluated and approved by the responsible independent ethics committees. The principal investigator will notify the participating centres and send a copy of the revised protocol to the responsible investigator to add to the Investigator Site File. Furthermore, an update of the trial register will be initiated.

#### Eligibility

##### Inclusion criteria

All patients scheduled for elective RAMIE for oesophageal cancer with intrathoracic oesophagogastrostomy (Ivor-Lewis) may be included in this study. Further inclusion criteria are as follows: patients of any sex, age ≥ 18 years, American Society of Anaesthesiologists (ASA) score ≤ III, Eastern Cooperative Oncology Group (ECOG) status ≤ II, suitability for both management strategies, ability to understand the characteristics, and individual consequences of the clinical trial and written informed consent.

##### Exclusion criteria

The exclusion criteria are as follows: open oesophagectomy (either abdominal or thoracic), emergency operations, chronic pain syndromes requiring routine analgesics, simultaneous lung resection, or major lung laceration that has been sutured or stapled. Furthermore, the presence of contraindications to the use of epidural anaesthesia (e.g. coagulopathies, anticoagulation disorders, or allergies), participation in a competing interventional trial and impaired mental state will prohibit inclusion.

### Withdrawal criteria

Trial subjects may withdraw from the trial at their request or if RAMIE is not performed (e.g. due to technical irresectability, conversion to conventional resection, or metastatic disease). However, patients’ withdrawal and reasons will be described by a CONSORT flow diagram to ensure full transparency.

### Interventions

#### Surgical technique

To avoid the risk of learning-associated bias, only surgeons with sufficient proficiency will be allowed to perform the randomized interventions. Surgeons involved in the trial as the responsible surgeons must have a personal operative experience of at least 20 RAMIEs. The standard technique and surgical instruments may vary in several aspects. The technique of the Dresden Center was described elsewhere [16–18]. Guidelines for the present study are as follows:

- The abdominal part can be performed either laparoscopically or robotically.
- Intraoperative abdominal drains can be placed through the trocar sites in both groups.
- The patients are then placed in a left lateral decubitus position.
- The thoracic part is performed robotically in all cases. A circular, end-to-side, oesophagogastrostomy after gastric tube pull-up is performed over muscle-sparing mini-thoracotomy to reduce postoperative pain.
- Randomization is performed intraoperatively after the completion of the oesophagogastrostomy.
- Two 24 Charrière (Ch) chest drains are inserted through trocar sites robotic arms 1 and 3 (R1 and R3) at the end of the procedure in the control group; one 24 Ch chest drain is inserted through trocar site robotic arm 3 (R3) in the intervention group. The chest drains are immediately connected to the vacuum suction device to remove the remaining postoperative air in the right pleural cavity.
- Muscles at the trocar sites will be sutured with resorbable material (e.g. Vicryl 2–0 or equivalent) in a single stitch technique.
- Chest drains will be secured with a non-resorbable suture anchored to the skin. This should prevent excessive travel of the drain in and out of the chest wall. The suture will be looped several times around the drainage to achieve airtight wound closure after the drainage has been removed by knotting the suture. The skin incision can be closed on each side of the chest tube, usually with additional sutures if necessary. Further skin incisions will be closed with resorbable sutures (3–0 or equivalent) or staplers.
- The patient is turned into a supine position for extubation or transferal to the intensive care unit.
- In the intervention group, abdominal drains (if inserted) and chest drains (if air leak is < 100 ml/min, not increasing over 2 min, pneumothorax < 2.5 cm and no signs of active bleeding are present) are removed after chest X-ray 3 h after the end of surgery.
- In the control group, abdominal drains are removed after 3 h, and the two chest drains remain in place (suction − 15 mmHg). These drains are removed during further postoperative days according to a standard algorithm (see below).
- According to the standard algorithm, a chest X-ray is performed on POD1 and POD 3. The first chest tube can be removed at POD 3 if the daily secretion is below 450 ml per 24 h and if there is no evidence of an air leak (< 100 ml/min), pneumothorax > 2.5 cm, chyle leak, purulent secretion or bleeding followed by chest X-ray for control. On POD 6, chest drain 2 is removed if the daily secretion is below 450 ml per 24 h, followed by radiographic control. Deviations because of clinical conditions or logistic delays are allowed.

#### Perioperative pain management

The thoracic epidural catheter is placed preoperatively (before induction of anaesthesia) at the vertebral level of Th 6–9. After a test dose with 3.5 ml 1% prilocaine to exclude intraspinal placement, epidural regional anaesthesia will be started with a bolus injection of 10 ml ropivacaine hydrochloride 0.3% and 20 µg sufentanil after induction of anaesthesia and at least 20 min before skin incision. Immediately afterwards, a continuous infusion of 5 ml/h ropivacaine hydrochloride 0.2% will be maintained for at least 2 days. Additional epidural boluses of 5 ml are allowed every 20 min at the discretion of the anaesthesiologist during the operation and according to the need of the patient postoperatively. After the initial 200-ml bag of ropivacaine hydrochloride, 0.2% plus 0.5 µg/ml sufentanil is empty, the replacement will be done without sufentanil, with pure ropivacaine hydrochloride 0.2%. Postoperatively, the concomitant pain medication will consist of 1 g of metamizole or 1 g of paracetamol 4 times daily. That can be extended to 10 mg extended-release oxycodone (every 12 h) and an on-demand medication of 10 mg immediate-release oxygesic if necessary, in case of a persistent pain score > 4 according to the numeric rating scale (NRS).

The running rate and dosage will be checked daily by the pain service and adjusted to the individual needs of the patient (including increasing the basal rate to a maximum of 8 ml/h, reducing/terminating the basal rate of continuous epidural infusion and removal of the catheter).

### Assignment and randomization

All patients will be screened for eligibility considering the inclusion criteria on the day of admission (standard: the day before the surgery). Screened and eligible patients will be included in the trial after obtaining written informed consent. After obtaining written informed consent, randomization will be performed intraoperatively after completion of the oesophagogastrostomy and verification of the exclusion criteria. To achieve comparable intervention groups for known and unknown risk factors, randomization will be performed using block randomization with variable block length. Randomization lists are stratified by trial sites and will be centrally generated by nQuery by the Coordination Centre for Clinical Trials. A patient will be randomized by authorized trial personnel using the electronic REDCaP® system.

### Objectives

#### Primary endpoint

Chest drains can cause postoperative pain by irritating intercostal nerves and compromising pulmonary function independent of the surgical approach [19]. Pain impairs early mobilization. Both postoperative immobility and limited pulmonary function may further exert deleterious effects on the postoperative clinical course. Therefore, the objective is to evaluate potential short-term advantages between RAMIE without abdominal and early removal of chest drains compared with the postoperative use of chest drains. The primary hypothesis is that the early removal of chest drains after RAMIE reduces pain in the early postoperative period compared with RAMIE with drains, which is currently considered standard. Postoperative pain according to a numeric rating scale (NRS) on the second postoperative day (POD) was chosen as the primary study endpoint.

#### Secondary endpoints

The secondary endpoints reflect important treatment outcomes and thus enable a comprehensive evaluation of the surgical therapy and the postoperative course of treatment.

Perioperative complications associated with oesophagectomy are defined and recorded according to the Oesophagectomy Complications Consensus Group (ECCG) definition [20]. The secondary endpoints are pain according to NRS in the period from POD 1 to 7 (daily, Table 1), mean postoperative pain (NRS) on POD 2–4, additional (> standard) analgesic drug use during POD 2–4, drain secretion volumes, operating time, blood loss, conversion rate, number of postoperative chest X-ray and computed tomography (CT) scans, interventions including chest CT drain or chest tube replacement, postoperative mobilization (steps per day as measured with an activity tracker), and time to functional recovery. Time to functional recovery will be defined as the postoperative time point at which the following criteria are fulfilled: absence of all wound/chest drains and no requirements of intravenous analgesics and tolerance of oral intake and ability to mobilize independently.

**Table 1:** Determination of postoperative pain during study visits

Abdominal pain?	NRS 0-10
Chest pain?	NRS 0-10
Others?	NRS 0-10
Pain on exertion?	NRS 0-10
Maximum pain since surgery?	NRS 0-10
Minimal pain since surgery?	NRS 0-10
Is your mobility or movement impaired by the pain?	Yes/no
Is the pain affecting your ability to cough or breathe deeply	Yes/no
Did the pain wake you up during the night	Yes/no
Is your mood affected by the pain?	Yes/no
Have you felt very tired since the operation?	Yes/no
Have you suffered from nausea since the operation?	Yes/no
Have you vomited since the operation?	Yes/no
Would you have liked to have more painkillers received?	Yes/no
How satisfied are you with the pain treatment you have received since the operation?	0–10°

0 = very dissatisfied; 10 = very satisfied

Furthermore, oesophagectomy-associated postoperative morbidity (pneumothorax, pleural effusion, chylothorax, pneumonia, anastomotic leakage, surgical site infection), in-hospital mortality, length of intensive care unit (ICU) stay, and hospital stay will be analysed. Postoperative morbidity from index surgery to discharge will be assessed using the comprehensive complication index based on the Clavien‒Dindo classification.

### Trial visits

The trial includes a total of nine study visits during the operation and the postoperative period. The inclusion and exclusion criteria will be evaluated during the screening visit (the day before the operation). After the patient has given his or her informed consent, the demographic and baseline data, medical history, current medication, comorbidities, American Society of Anaesthesiologists (ASA) classification, and ECOG status will be evaluated and documented. Randomization will be performed intraoperatively by authorized study personnel, and intraoperative parameters will be collected.

Patients are followed up until discharge. Study visits will be scheduled on postoperative days 1 to 7 and on the day of discharge from the hospital. There will also be a daily visit by the pain service until the epidural catheter is removed. Primary and secondary endpoints and (serious) adverse events (SAEs and AEs) will be evaluated and documented during each visit and documented on the case report form (CRF). All planned study visits are summarized in Table 2. Data from patients without RAMIE (conversion, inoperability) will be documented until the time of the operation (visit 1). Routine blood tests (including haemoglobin concentration, leucocyte count, and serum C-reactive protein) will be performed during the postoperative visits on PODs 2, 4, and 6. In addition, postoperative chest X-rays are performed routinely on postoperative days 1, 3, and 6 independent of the study intervention or additionally according to the clinical course. According to the protocol, a CT scan of the thorax or abdomen or diagnostic endoscopy will not be routinely performed during the study period. These diagnostic examinations are only indicated based on a medical rationale (e.g. control scan of pneumothorax before or after chest drain removal, suspected fluid collection or abscess, elevated C-reactive protein or leucocyte count, suspicion of anastomotic leakage).

**Table 2:** Study visits

Documentation	Screening day of admission	Day of operation	PODs 1–7 or until epidural catheter is removed	Day of discharge
Inclusion criteria	X			
Exclusion criteria	X	X		
Patient characteristics	X			
Randomization		X		
Surgical technique		X		
NRS pain scale			X	
Morbidity			X	X

### Assessment of safety

Adverse (AEs) and serious adverse events (SAEs) will be recorded during this trial.

An SAE is defined as any life-threatening adverse event, which requires or prolongs hospital stay, results in persistent or significant disability or incapacity, or results in death. Events occurring in the period from randomization to discharge must be documented and reported to the coordinating investigator within 5 days after they become apparent. All SAEs must be documented on a “serious adverse event form”.

The form contains the following information: name of the attending physician, detailed description of the SAE, consequence for the trial, and dated signature of the attending physician.

### Statistics

#### Statistical considerations and sample size calculation

A worldwide accepted practice after open or minimally invasive oesophagectomy is to place apical and basal chest drains for complete drainage of the pleural cavity. The sample size calculation is based on a comparison of double versus single chest drain applications after pulmonary lobectomies. In a prospective randomized trial, the mean values of postoperative pain according to the visual analogue scale (VAS) on the second postoperative day were 4.28 in the single-drain group *versus* 5.10 in the double-drain group (*p* = 0.001). Furthermore, the authors described a significant reduction in the amount of drainage fluid (600 ml vs. 896 ml). The authors concluded that the insertion of two chest tubes is not more effective than a single chest tube after lobectomy. In addition, the absence of chest tubes causes significantly less postoperative pain [21, 22]. These results are supported by the results of another study group that found a significant difference in the maximum postoperative pain score when comparing one with two postoperative chest drains [21, 22]. The sample size estimation for the present trial was therefore based on a reduction of pain scale measured by NRS from five points in the group with drains to four points in the intervention group on POD 2 and assuming an equal standard deviation of 1.4 for both groups. To achieve 80% power with a two-sided *t*-test for two independent groups and a significance level of 5%, a group size of 32 patients is necessary. With an estimated dropout rate of 10%, the total sample size was calculated with 72 patients with 36 in each of the two groups. Sample size calculation was performed using the SAS 9.4 software (Cary, NC 27,513–2424, USA). Confirmatory analysis of the primary endpoint will be carried out by a two-sided *t*-test for independent groups on a modified intention-to-treat population, which comprises all patients randomized with successful RAMIE. Statistical significance will be set at 0.05. Secondary endpoints will be analysed by Fisher’s exact test, Student’s *t*-test, Mann–Whitney *U* test, mixed models, and Kaplan‒Meier method, as appropriate. Sensitivity analyses will be conducted on the per-protocol set. Statistical analyses will be performed using the R statistics software package (version 3.1.3 “or higher”, the R Foundation for Statistical Computing). No interim analysis of primary or secondary endpoints of efficacy is planned.

### Blinding

Blinding of participants, research assistants, operating surgeons, data collectors, and outcome assessors to the treatment allocation is not feasible. Postoperative blinding of patients and outcome assessors is not possible since unblinding would occur when vacuum suction was removed before the assessment of the primary endpoint. Outcome assessment will be carried out by trained study personnel who are not part of the surgical or ward team to guarantee objectivity. Data analysts will be blinded to the intervention.

### Data processing

#### Patient education and written informed consent

All consecutive patients were screened for potential inclusion by the investigator at the respective participating centres. Eligible patients are asked for informed consent. Before randomization, each patient will be informed by authorized investigators about the nature, objectives, expected benefits, and potential risks of the trial. Each patient must declare his or her willingness to participate in the study verbally and in writing after being informed in a manner that he or she can understand. Each patient must be informed that he or she is allowed to withdraw their informed consent verbally or in written form at any time without receiving any reprisals or disadvantages. In the event of revocation of the declaration of consent, the data stored up to this point in time will continue to be used without naming a name if this is necessary to evaluate the effects of the investigational product and to ensure that the interests of the person concerned worthy of protection are not impaired.

### Data collection

After the patient gave his or her informed consent, the demographic and basic data (sex, age, weight, height, medical history, current medication, comorbidities, ASA classification, ECOG status), and primary tumour characteristics (UICC classification, date of diagnosis, location, previous treatments including chemotherapy, target therapy, radiotherapy, and surgery) are evaluated and documented by the investigator. Intraoperative parameters such as length of operating time, blood loss, blood transfusion requirement, and conversion to open surgery are recorded.

During each study visit and on the day of discharge, primary and secondary endpoints and SAEs are evaluated and documented according to the eCRF. During the postoperative visits, routine blood tests (including haemoglobin concentration, leucocyte count, and serum C-reactive protein) will be performed. Furthermore, data that will be collected within these study visits include at least the following: postoperative assessment, ICU length of stay, grading of complications according to Clavien‒Dindo Classification, morbidity and mortality within the hospital stay, length of hospital stay, drain secretion volumes, postoperative examinations (e.g. chest X-rays, CT scans), and physical activity of the patient. The physical activity of the patients is collected using a CE-certified fitness tracker device, which measures the number of steps, distance, and activity minutes during the hospital stay. The transmission of these data works automatically via synchronization with a tablet or is carried out by the investigators before discharge.

### Documentation

It is the responsibility of the investigator to ensure that the study is conducted in accordance with the professional code for physicians, the Declaration of Helsinki in its current version, and the study protocol and that the data are properly documented. All data collected in this study must be entered into the eCRF by appropriately authorized persons. This also applies to data from persons who have been excluded from the study. The study site records participation on a patient identification list. This list is used for the identification of the participating persons and contains the patient number, full name, date of birth, and date of admission to the RESPECT trial. The patient identification list remains at the trial centre after the study is completed.

Using the audit trail in the REDCaP® online database, all data and corrections are automatically logged with the date, time, and the person making the entry so that former entries can be retrieved at any time.

Records and documents related to the RESPECT trial (e.g. documentation of informed consent, worksheets, and other relevant documents) must be retained by the investigator for 10 years in accordance with Good Clinical Practice.

### Data management and quality assurance

Day-to-day support for the trial is provided by the principal investigator who will take supervision of the trial and the medical responsibility. All data management will be performed in the REDCaP® online database. The entered data are managed and processed by the study centre of the Department of Visceral, Thoracic, and Vascular Surgery, University Hospital Carl Gustav Carus, TU Dresden, the Department of General, Visceral, and Transplantation Surgery, Heidelberg University Hospital and further participating centres in accordance with data protection regulations.

The data are checked by range, validity, and consistency checks. The site staff is responsible for data correction. A statement must be provided for any missing data.

The data manager will supervise and support the solution of data discrepancies and will close all correctly resolved discrepancies. If an outstanding entry cannot be solved, the data manager may close the entry. At the end of the study, the database will be closed after all relevant data according to the study protocol have been entered. Thereafter, any changes to the database are possible only by a joint written agreement between the coordinating investigators and the data manager.

#### Data Monitoring Board (DMB)

In case of any irregularities, for example, concerning the prevalence or type of adverse events or serious adverse events reported to the principal investigator, the members of the independent DMB will be informed without any delay. The independent DMB will receive regular reports about major complications after 20 and 40 patients have been randomized. The results of the risk and safety analysis will be reported to the principal investigator, including recommendations concerning the continuation or termination of the trial.

Monitoring will be conducted according to approved standard operating procedures, which include personal site visits with verification of source data. Clinical monitoring in the RESPECT study will be performed according to the International Conference of Harmonization (ICH) Good Clinical Practice (GCP) guidelines by the Study Center of the Department of Visceral, Thoracic, and Vascular Surgery of the University Hospital and Medical Faculty Carl Gustav Carus, Dresden. The monitoring procedures are predefined in a study-specific monitoring manual and adapted to the study-specific risks for the patients. Regular on-site monitoring visits are planned at all sites depending on the recruitment rate and quality of the data (approximately one visit per site and year). The investigator must allow the monitor to look at all essential documents and must always provide support to the monitor. Study progress, recruitment, and data capture will also be reviewed at the leading centre by the principal investigator and study coordinators.

The role of the Steering Committee is to provide overall oversight of the trial and ensure that it is conducted according to the rigorous standards as outlined in the Guidelines for Good Clinical Practice.

### Ethical considerations

According to the Medical Association’s Professional Code of Conduct “Ärztliche Berufsordnung” of each participating German state, an independent Ethics Committee (IEC) must be consulted before the start of the clinical study concerning questions relating to professional ethics and legal issues, which are associated with the study. For this purpose, the study protocol, patient information sheet, and informed consent must be submitted to the responsible Ethics Committee.

The trial will be conducted in line with the Declaration of Helsinki in its current version and with the laws and declaration of the concerned country. This protocol is designed to ensure that the study is carried out and analysed according to ICH-GCP. The trial is in line with the Consort Guidelines [23].

## Discussion

In the past, the removal of chest drains after oesophageal resections were often determined by the daily amount of secretion. For many surgeons, a daily secretion of less than 200 ml in 24 h represented a cut-off value from which drainage could be safely removed. However, these values were based on prevailing doctrine and the concern of having to reinsert a chest drain rather than on a well-founded scientific data [14].

Enhanced recovery after surgery (ERAS) protocols after oesophagectomy can additionally lower morbidity but still include thoracic chest drains for a minimum of a week after the operation. Reduction in the number and early removal of chest drains has already been advocated after pulmonary resections.

The current data show that more aggressive chest drain removal strategies under ERAS programmes are safe and feasible. For example, a threshold of 500 ml per 24 h led to recurrent pleural effusion after video-assisted thoracic surgery in only 2.8% of patients [24]. However, at a daily secretion of 450 ml, removal of the chest drain was no longer associated with recurrent pleural effusion [25]. Therefore, the authors concluded that removal of the drain seems to be possible if there is no evidence of air leakage, pneumothorax, chyle leak, purulent secretion, or active bleeding.

Regarding the number of chest drains, several studies have shown that the insertion of two chest tubes is not superior to the insertion of a single chest tube after pulmonary lobectomy. The use of one chest tube is more effective than the use of two tubes because it causes less postoperative pain, less pleural effusion, and reduced chest tube duration without affecting postoperative morbidity [22, 24, 26]. Referring exclusively to postoperative pain, the data show a significant reduction between two and one chest drain on the second day after surgery [21, 22, 26]. In addition, one study revealed a significant decrease in the course of the second postoperative week [22].

There are no reports of randomized controlled trials investigating the potential advantages of modern RAMIE for oesophageal cancer without postoperative abdominal and chest drains compared to the postoperative use of chest drains, which is considered standard practice worldwide. This randomized controlled multicentric trial was therefore initiated to assess whether avoidance of chest drains during the postoperative course after RAMIE can significantly reduce postoperative pain, improve functional recovery, and shorten hospital stay compared to the use of chest drains. Avoidance of chest drains during the postoperative course is achieved by the removal of a chest drain 3 h after the end of the surgical procedure. The current standard in consensus fast-track protocols after oesophagectomy is the removal of chest tubes on POD 6 [13, 21]. The 3-h period is necessary and sufficient to enable complete expansion of the right lung and to exclude a persistent air leak. This concept has been successfully applied in daily practice after RAMIE in selected cases at the authors’ institution. Its implementation in everyday use would first and foremost be a step forwards for our patients. The healthcare system would benefit from shorter hospital stays and associated cost savings. Finally, it would be a step forwards for surgical science because the reduction of postoperative morbidity is both a key aim and a major driver of surgical innovation.

This study aims to compare two different chest drain management strategies in patients undergoing RAMIE for oesophageal cancer with regard to perioperative complications until discharge. The study’s primary objective is to investigate whether the intensity of postoperative pain can be significantly reduced by avoiding thoracic drains after RAMIE. We assume that this will influence secondary endpoints such as early recovery and length of hospital stay.

## Trial status

### Recruiting

The first patient will be enrolled in March 2023. It is anticipated that recruitment will finish in October 2024.

Issue V 2.0: 21.03.2022.

## Data Availability

The participant information materials and informed consent form are available from the corresponding author on request.

## Abbreviations

ASA: American Society of Anaesthesiologists
eCRF: Electronic case report form
ERAS: Enhanced recovery after surgery
RAMIE: Robot-assisted minimally invasive oesophagectomy
POD: Postoperative day
